# Supplementary material for: The role of cytochrome bc1 inhibitors in future tuberculosis treatment regimens
Source: Nat Commun. 2025 Oct 22;16:9344. doi: 10.1038/s41467-025-64427-6 (PMC12546632; doi:10.1038/s41467-025-64427-6)
Supplement: Supplementary file 1 — Supplementary Information [file 41467_2025_64427_MOESM1_ESM.pdf]

SUPPLEMENTARY INFORMATION

The role of cytochrome bc_1 inhibitors in future tuberculosis treatment regimens

Clara Aguilar-Pérez[1,*], Anne J. Lenaerts[2], Cristina Villellas[1], Jerome Guillemont[3], John Dallow[4], Hannah Painter[4], Nicole C. Ammerman[5,6], Anis Hassan[4], Guillaume Golovkine[7], Laure Brock[7], Sylvie Sordello[7], Aurélie Chauffour[8], Alexandra Aubry[9], Thi Cuc Mai[8], Sarah Wong[8], Taane G. Clark[4], Kiyean Nam[10], Jeongjun Kim[10], Jinho Choi[10], Marjolein Crabbe[11], Jorge Esquivias[12], Nacer Lounis[1], Bart Stoops[11], Katie Amssoms[11], Jose M. Bartolome-Nebreda[12], Veronica Gruppo[2], Gregory T. Robertson[2], Nicolas Veziris[9], Anna M. Upton[13], Eric L. Nuermberger[5], Vivian Cox[14], Lluis Ballell[12], Benny Baeten[1], Anil Koul[1,4], Alexander S. Pym[15], Richard J. Wall[4,#], Dirk A. Lamprecht[1,16,*,#]

[1]Janssen Global Public Health, LLC, Janssen Pharmaceutica NV, Turnhoutseweg 30, 2340 Beerse, Antwerpen, Belgium

[2]Mycobacteria Research Laboratories, Department of Microbiology, Immunology and Pathology, Colorado State University, Fort Collins, Colorado 80521, USA

[3]Janssen Infectious Diseases Discovery, Janssen-Cilag, Val de Reuil, France.

[4]Department of Infection Biology, Faculty of Infectious and Tropical Disease, London School of Hygiene and Tropical Medicine, London, WC1E 7HT, UK

[5]Center for Tuberculosis Research, Department of Medicine, Johns Hopkins University, Baltimore, MD, USA

[6]Erasmus MC, University Medical Center Rotterdam, Department of Medical Microbiology and Infectious Diseases, Rotterdam, Netherlands

[7]Translational Biology, Infection Diseases, Evotec, 195, Route D'Espagne, 31100, Toulouse, France

[8]Sorbonne Université, INSERM, Centre d'Immunologie et des Maladies Infectieuses, U1135, Paris, France

[9] Sorbonne Université, INSERM, Centre d'Immunologie et des Maladies Infectieuses, U1135, APHP Sorbonne Université, Centre National de Référence des Mycobactéries et de la Résistance des Mycobactéries aux Antituberculeux, Paris, France

[10]Qurient Co. Ltd. C-dong 801, 242, Pangyo-ro, Bundang-gu, Seongnam-si, Republic of South Korea

[11]Janssen Research & Development, LLC, Janssen Pharmaceutica NV, Turnhoutseweg 30, 2340 Beerse, Antwerpen, Belgium

[12] Discovery Chemistry, Janssen-Cilag SA a Johnson & Johnson company, C. Río Jarama, 75A, 45007 Toledo, Spain

[13]Evotec US inc., 303B College Road East, Princeton, New Jersey, 08540, USA.

[14]Johnson & Johnson Innovative Medicine, Titusville, New Jersey, USA

[15]Janssen Global Public Health, LLC, Janssen Pharmaceutica, 50-100 Holmers Farm Way, High Wycombe, HP12 4DP, UK

[16]Current address: Holistic Drug Discovery and Development (H3D) Centre, University of Cape Town, Rondebosch 7700, South Africa

[#]These authors contributed equally.

Supplementary Figures

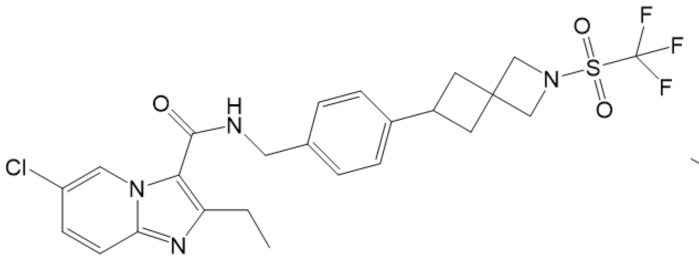

JNJ-2901

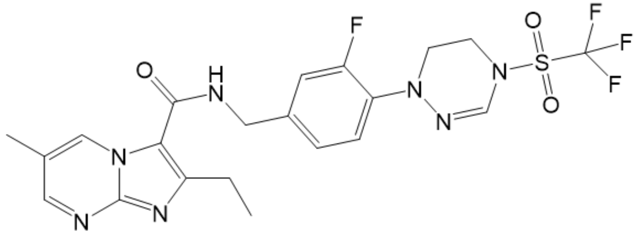

JNJ-4052

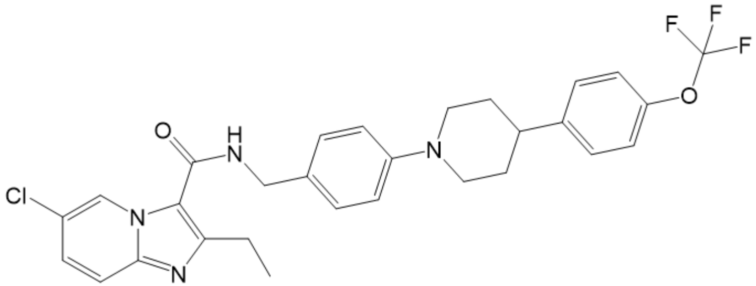

Q203 (Telacebec)

Supplementary Fig. 1: Chemical structure of Q203 (Telacebec)[1] and tool compounds JNJ-2901 and JNJ-4052

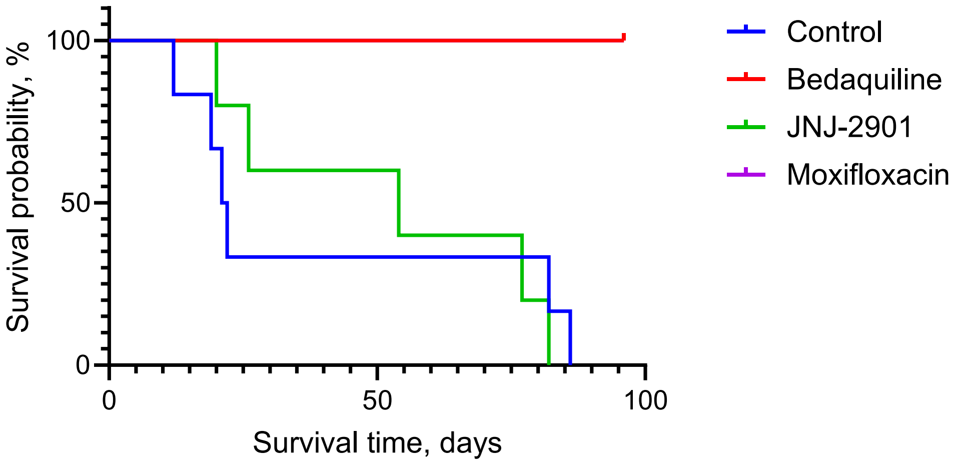

Supplementary Fig. 2: Survival probability of monotherapy treatment in intravenous model (IV) - Related to Study C. Six mice per group were infected with 6.1 $\log_{10}$ CFUs and, after 14 days, treated for three months (5 days per week) with monotherapy of either bedaquiline (25 mg kg^{-1}), moxifloxacin (100 mg kg^{-1}) or JNJ-2901 (10 mg kg^{-1}); 6 mice were left untreated. Bedaquiline and moxifloxacin overlap due to 100% survival observed in both conditions.

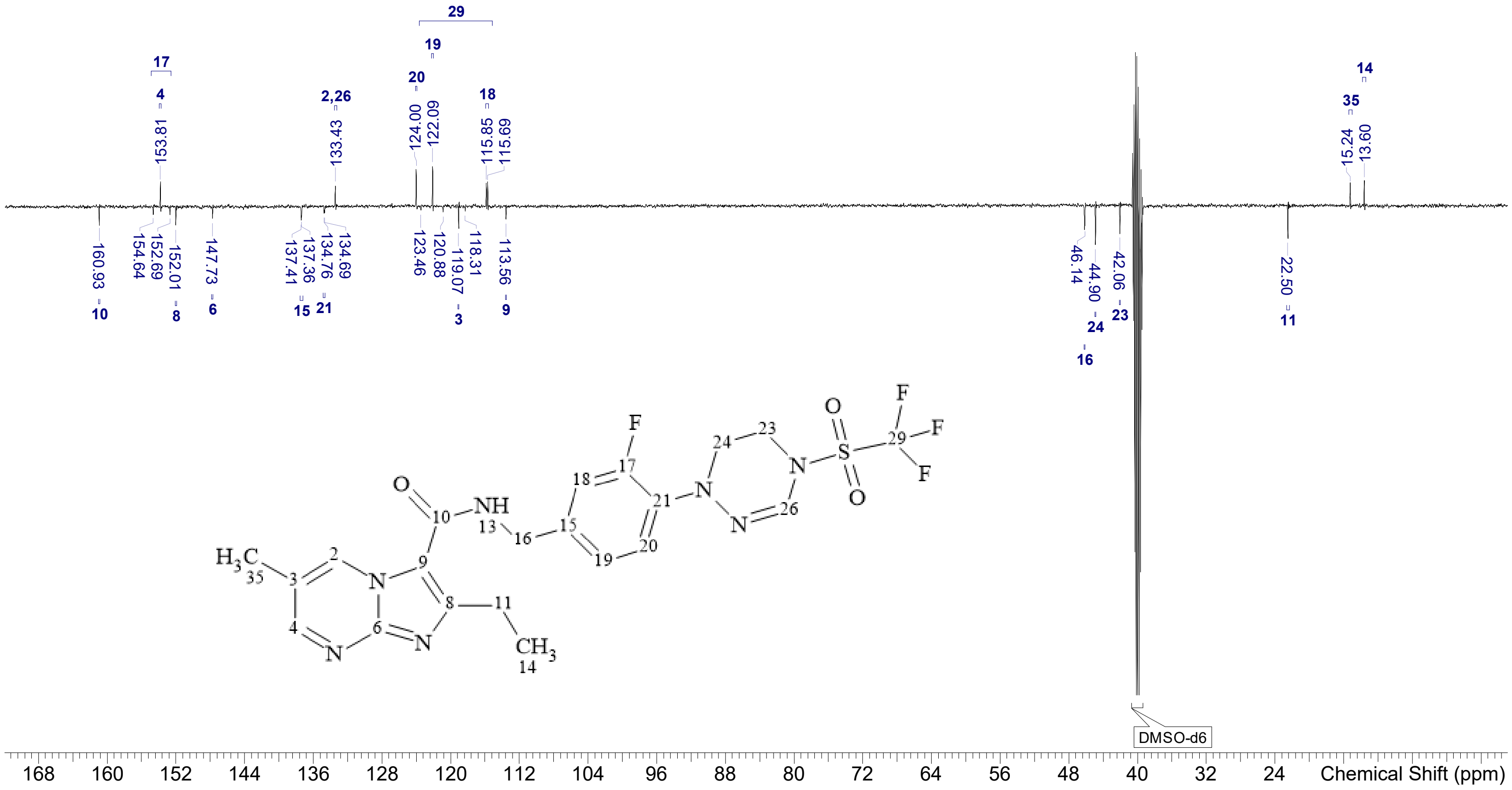

^{13}C NMR (125.76 MHz, DMSO-d_6) δ ppm 160.93 (s, 1 C) 153.81 (s, 1 C) 153.67 (br d, *J*=245.43 Hz, 1 C) 152.01 (s, 1 C) 147.73 (s, 1 C) 137.38 (br d, *J*=6.43 Hz, 1 C) 134.72 (br d, *J*=9.19 Hz, 1 C) 133.43 (s, 2 C) 124.02 (d, *J*=3.68 Hz, 1 C) 122.10 (d, *J*=1.84 Hz, 1 C) 119.07 (s, 1 C) 115.77 (d, *J*=20.22 Hz, 1 C) 119.59 (q, *J*=323.00 Hz, 1 C) 113.56 (s, 1 C) 46.16 (br d, *J*=5.52 Hz, 1 C) 44.90 (s, 1 C) 42.06 (s, 1 C) 22.50 (s, 1 C) 15.24 (s, 1 C) 13.60 (s, 1 C)

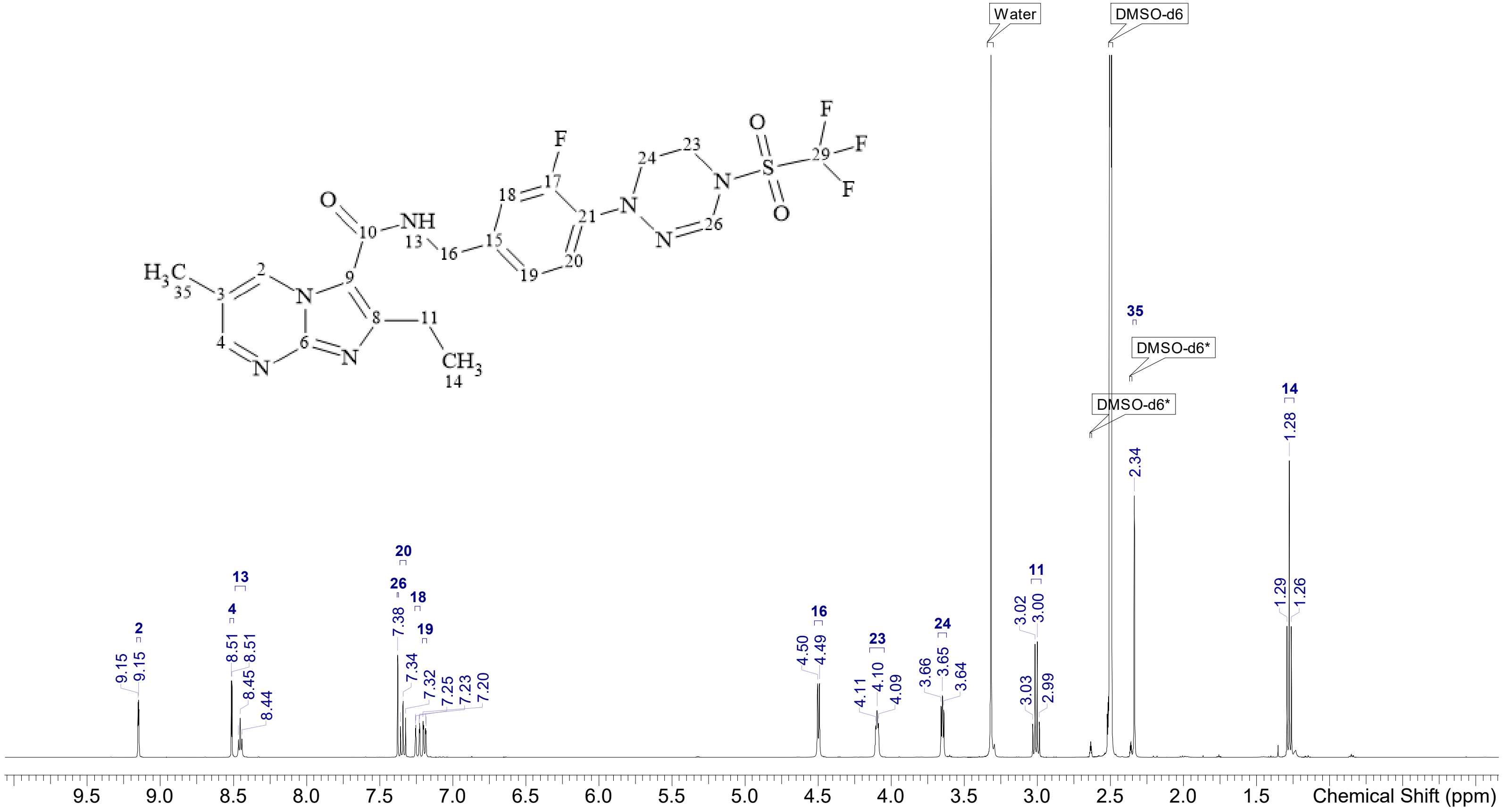

^{1}H NMR (500 MHz, DMSO-d_6) δ ppm 9.15 (dd, *J*=2.4, 1.1 Hz, 1 H) 8.51 (d, *J*=2.5 Hz, 1 H) 8.45 (t, *J*=5.9 Hz, 1 H) 7.38 (s, 1 H) 7.34 (t, *J*=8.6 Hz, 1 H) 7.24 (dd, *J*=13.2, 1.8 Hz, 1 H) 7.19 (dd, *J*=8.4, 1.8 Hz, 1 H) 4.50 (d, *J*=5.9 Hz, 2 H) 4.10 (t, *J*=4.6 Hz, 2 H) 3.62 - 3.68 (m, 2 H) 3.01 (q, *J*=7.5 Hz, 2 H) 2.34 (d, *J*=0.8 Hz, 3 H) 1.28 (t, *J*=7.5 Hz, 3 H)

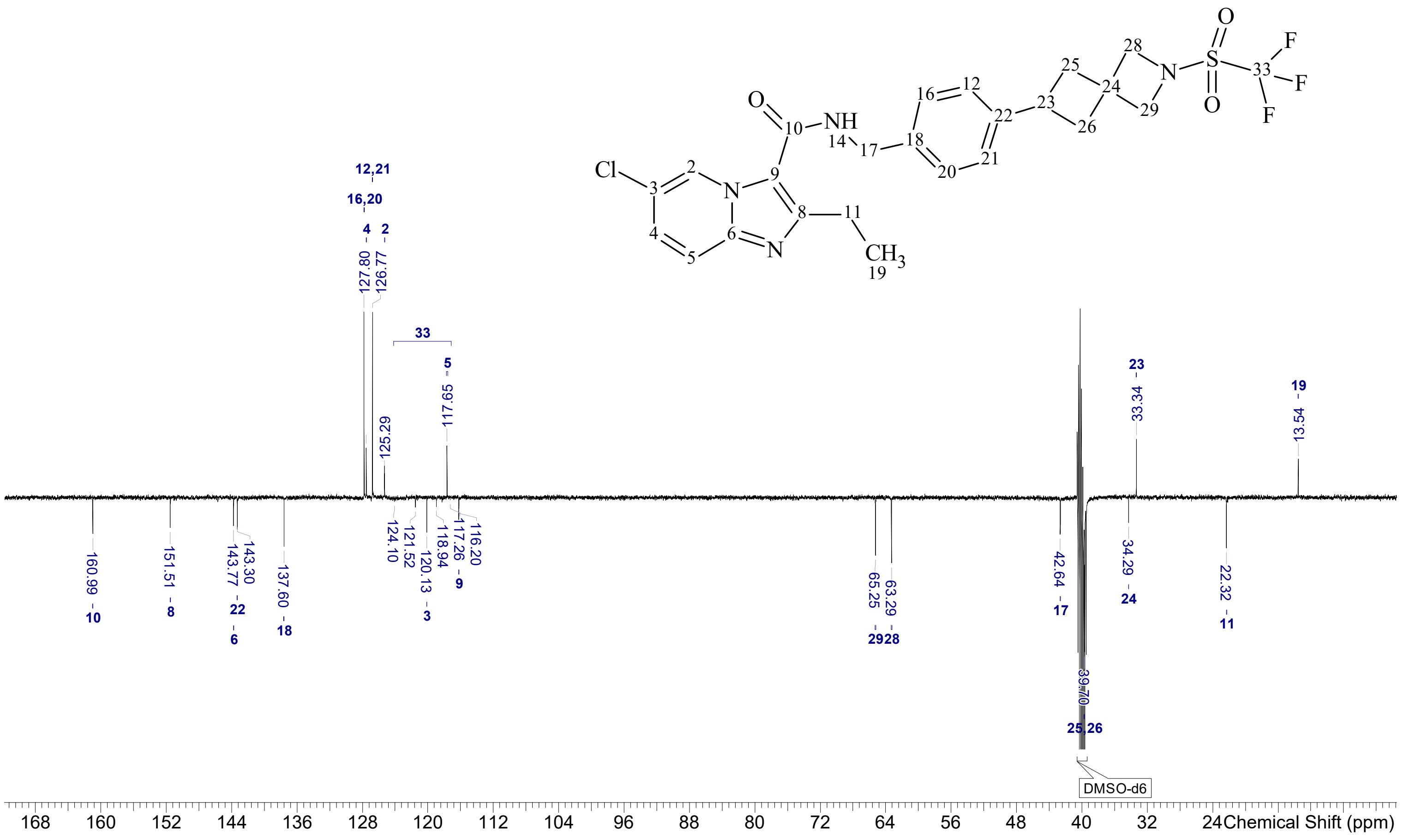

^{13}C NMR (125.76 MHz, DMSO-d_6) δ ppm 160.99 (s, 1 C) 151.51 (s, 1 C) 143.77 (s, 1 C) 143.30 (s, 1 C) 137.60 (s, 1 C) 127.80 (s, 2 C) 127.52 (s, 1 C) 126.77 (s, 2 C) 125.29 (s, 1 C) 120.13 (s, 1 C) 117.65 (s, 1 C) 120.62 (q, J=323.00 Hz, 1 C) 116.20 (s, 1 C) 65.25 (s, 1 C) 63.28 (d, J=4.60 Hz, 1 C) 42.64 (s, 1 C) 39.70 (s, 2 C) 34.29 (s, 1 C) 33.34 (s, 1 C) 22.32 (s, 1 C) 13.54 (s, 1 C)

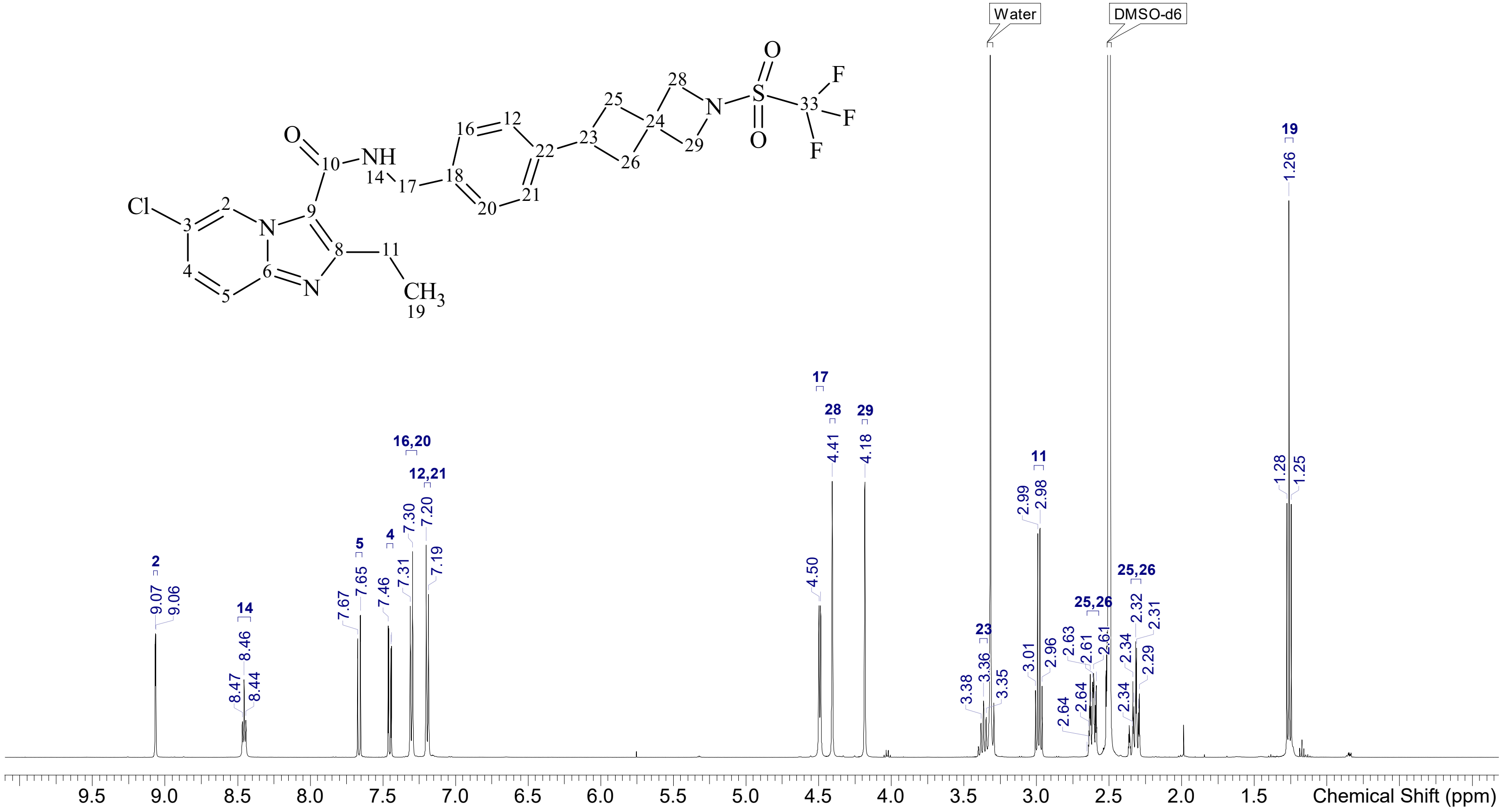

^{1}H NMR (500 MHz, DMSO-d_6) δ ppm 9.07 (dd, *J*=2.1, 0.7 Hz, 1 H) 8.46 (t, *J*=6.1 Hz, 1 H) 7.66 (dd, *J*=9.5, 0.9 Hz, 1 H) 7.45 (dd, *J*=9.5, 2.1 Hz, 1 H) 7.30 (m, *J*=8.1 Hz, 2 H) 7.19 (m, *J*=8.1 Hz, 2 H) 4.49 (d, *J*=5.9 Hz, 2 H) 4.41 (s, 2 H) 4.18 (s, 2 H) 3.34 - 3.39 (m, 1 H) 2.98 (q, *J*=7.5 Hz, 2 H) 2.57 - 2.65 (m, 2 H) 2.28 - 2.35 (m, 2 H) 1.26 (t, *J*=7.5 Hz, 3 H)

Supplementary Fig. 3: ^{1}H and ^{13}C NMR spectra for JNJ-2901 and JNJ-4052

Supplementary Tables

Supplementary Table 1: Profiling of JNJ-2901- and JNJ-4052-resistant strains against other cytochrome *bc*$_1$ inhibitors and selected clinical compounds – MIC$_{50}$ values (µM) against other compounds from the series and clinical compounds with fold difference from the starting parental strain shown in brackets**.** Resistance strains were generated in a cytochrome bd knockout (Δ*cydAB*; Rv1622 and Rv1623) background in the presence of 100x MIC$_{90}$ of JNJ-2901 and JNJ-4052. Key resistance mutations shown for each strain.

	**MIC$_{50}$, µM (fold change)**							
	**Parent** (Δ*cydAB*)	**JNJ-2901-R1** QcrB; A317V	**JNJ-2901-R2** QcrA; L356W	**JNJ-4052-R1** QcrB; A317T	**JNJ-4052-R2** QcrA; L356W	**JNJ-4052-R3** QcrB; A317T	**JNJ-4052-R4** QcrB; T313A	**JNJ-4052-R5** QcrB; A317V
**JNJ-2901**	0.01	1.02 (160)	1.02 (159)	0.05 (7)	1.05 (165)	0.06 (10)	2.99 (469)	0.93 (146)
**JNJ-4052**	0.01	19 (2717)	2.3 (324)	0.41 (57)	2.46 (344)	0.51 (71)	14 (2067)	6.14 (860)
**Q203**	0.01	0.75 (148)	0.84 (165)	0.03 (6)	0.38 (75)	0.04 (8)	1.06 (209)	0.66 (131)
**BDQ**	0.05	0.04 (1)	0.03 (1)	0.02 (0.5)	0.04 (1)	0.04 (1)	0.10 (2)	0.02 (0.5)
**RIF**	0.1	0.11 (1)	0.03 (0.5)	0.02 (0.25)	0.04 (0.5)	0.06 (1)	0.12 (1)	0.01 (0.25)
**INH**	0.51	0.46 (1)	0.54 (1)	0.27 (0.5)	0.32 (1)	0.36 (1)	0.60 (1)	0.56 (1)

Supplementary Table 2: *In vitro* ADME and toxicology profiles for JNJ-2901 and JNJ-4052. – LM Cl_{int}: liver microsome intrinsic clearance; Hep Clint: hepatocyte intrinsic clearance. ND: Not determined.

	Parameter	JNJ-2901	JNJ-4052
**Biology activity**	**MIC_{90} (H37Rv,** µM)	0.009	0.007
	**MIC_{90} (H37Rv_CydBd-KO,** µM)	0.006	0.007
	**$MBC_{99.9}$ (H37Rv,** µM)	>0.5	>1.25
	**$MBC_{99.9}$ (H37Rv_CytBd-KO,** µM)	0.015	0.3
**Toxicity**	**Cytotoxicity (HepG2, IC_{20} in** µM)	11.8	98.8
	**Mitotoxicity (Glu/Gal, IC_{50} in** µM)	>100/>100	>100/93.2
	Mutagenicity (AMES II)	negative	negative
**Plasma protein binding**	**Human** (% free)	0.003	1.46
	**Dog** (% free)	ND	3.43
	**Rat** (% free)	ND	2.67
	**Mouse** (% free)	ND	2.44
**Metabolic stability**	**Human Hep Cl_{int}** (µL min^{-1} 10^6 $cells^{-1}$)	10	6.7
	**Dog Hep Cl_{int}** (µL min^{-1} 10^6 $cells^{-1}$)	13	4.7
	**Rat Hep Cl_{int}** (µL min^{-1} 10^6 $cells^{-1}$)	16	19
	**Mouse Hep Cl_{int}** (µL min^{-1} 10^6 $cells^{-1}$)	15	12
	**Human LM Cl_{int}** (µL min^{-1} mg $protein^{-1}$)	63.3	20.4
	**Dog LM Cl_{int}** (µL min^{-1} mg $protein^{-1}$)	13.1	<7.7
	**Rat LM Cl_{int}** (µL min^{-1} mg $protein^{-1}$)	17.9	16.7
	**Mouse LM Cl_{int}** (µL min^{-1} mg $protein^{-1}$)	41.0	42
**Drug-drug interaction**	**3A4 inh., IC_{50} in µM**	>10	>20
	**2C19 inh., IC_{50} in µM**	>10	>20
	**2C8 inh., IC_{50} in µM**	>10	>20
	**2C9 inh., IC_{50} in µM**	>10	>20
	**2D6 inh., IC_{50} in µM**	>10	>20
	**1A2 inh., IC_{50} in µM**	>10	>20
	**Cyp3A4 ind. (%ctrl)**	1.25 µM = 3.1<br>10 µM = 11	1 µM = 3.0<br>10 µM = 21.2

Supplementary Table 3: Pharmacokinetic profiling of JNJ-2901 and JNJ-4052 - Results are expressed as the mean ± SD. n = 3 animals were dosed for the IV and PO arms and n = 3 animals by alternating sampling to limit the number of times blood was collected per animal.

**CD-1 mouse**				
	JNJ-2901		JNJ-4052	
**IV (1 mg kg^{-1})**	**PEG400:H_2O (70/30)**			
CLp (mL min^{-1} kg^{-1})	6.4 ± 1.6 (~9% LBF)		8.15 ± 0.82 (~11% LBF)	
Vss (L kg^{-1})	4.4 ± 0.8		1.80 ± 0.11	
$t_{½}$ (h)	12 ± 0.2		3.06 ± 0.51	
MRT (h)	9.1 ± 0.6		3.62 ± 0.52	
**PO (5 mg kg^{-1})**	**PEG400 (sol.)**	**0.5 w/v Methocel (susp.)**	**PEG400 (sol.)**	**0.5 w/v Methocel (susp.)**
C_{max} (ng mL^{-1})	474 ± 29	305 ± 59	1228 ± 406	787 ± 226
T_{max} (h)	7.0 (4.0-7.0)	4.0	4.0	2.0 (1.0-2.0)
AUC_{0-inf} (ng h mL^{-1})	9498 ± 1853	5287 ± 656	10880 ± 1715	5610 ± 2747
$t_{½}$ (h)	10	12 ± 3	3.55 ± 0.45	3.49 ± 0.91
F (%)	70 ± 14	N/A	106 ± 17	55 ± 27

**Balb/c mouse**				
	JNJ-2901		JNJ-4052	
**IV (1 mg kg^{-1})**	**PEG400:H_2O (70/30)**			
CLp (mL min^{-1} kg^{-1})			10.5 ± 1.3	
Vss (L kg^{-1})			2.24 ± 0.19	
$t_{½}$ (h)			2.33 ± 0.30	
MRT (h)			3.04 ± 1.01	
**PO (5 mg kg^{-1})**	**PEG400 (sol.)**	**0.5 w/v Methocel (susp.)**	**PEG400 (sol.)**	**0.5 w/v Methocel (susp.)**
C_{max} (ng mL^{-1})	2310 ± 767	2660 ± 469	1027 ± 39	1291 ± 121
T_{max} (h)	7.0 (4.0-7.0)	2.0	1.0 (0.5-1.0)	2.0
AUC_{0-inf} (ng h mL^{-1})	38500 (n=2)	28500 ± 935	7816 ± 660	8237 ± 1023
$t_{½}$ (h)	8.6 ± 2.8	5.9 ± 2.0	1.81 ± 0.17	2.00 ± 0.14
F (%)	N/A	N/A	98 ± 8	103 ± 13

Supplementary Table 4: Lung bacterial burden and percentage of relapse in *M. tuberculosis*-infected mice (Studies A and B) - BALBc female mice were infected intranasally with a high inoculum (4.5 log$_{10}$ CFU) of *M. tuberculosis* H37Rv. Beginning 2 weeks post-infection (pi) treatment was administered 5 days per week for 4, 8 and 12 weeks. Lung bacterial burden was assessed after 4 and 8 weeks of treatment (n=5 mice/group). Twelve weeks after 8 and 12 weeks of treatment, the proportion of mice with relapse was calculated (n=15 mice/group and n=30 mice/group [pooled data] (5/5)). D-13 = 1 day pi; D0 = day of treatment initiation, 2 weeks pi. Mice received different treatments combinations containing bedaquiline (B; 25 mg kg^{-1}), pretomanid (Pa; 40 mg kg^{-1}), linezolid (L; 100 mg kg^{-1}), clofazimine (C; 20 mg kg^{-1}) and JNJ-2901 (J; 5 mg kg^{-1}). *The proportion of mice with detectable CFU among all mice analysed was determined and expressed as % relapse. **Data are from Studies A and B. N, total number of mice/group; n, number of mice with CFU at plating. CFU, colony-forming unit; D, day; ND, not determined; SD, standard deviation; Tx, treatment; wks, weeks.

	**Mean ± SD log$_{10}$ CFU lung^{-1} and relapse (n/N)**							**relapse*, n/N (%)**		
**Treatment**	D-13		D0		4 wks Tx	8 wks Tx		8 wks Tx + (12 wks relapse)		12 wks Tx + (12 wks relapse)
**Study**	A	B	A	B	A	A	B	A	B	B
**Untreated**	4.46 ± 0.15	4.87 ± 0.09	7.23 ± 0.20	7.19 ± 0.32						
**BPaMZ**					1.25 ± 0.36 (5/5)	0.00 ± 0.00 (0/5)	ND	0/15 (0%)	ND	ND
**BPaL**					4.20 ± 0.25 (4/4)	2.00 ± 0.29 (4/4)	1.59 ± 0 (5/5)	13/15 (87%)	15/15 (100%)	4/15 (27%)
**BPaM**					4.36 ± 0.17 (5/5)	1.30 ± 0.43 (5/5)	2.77 ± 0.36 (5/5)	11/14 (79%)	14/14 (100%)	0/15 (0%)
**BPaMJ**					5.09 ± 0.23 (4/4)	2.96 ± 0.38 (5/5)	3.08 ± 0.53 (5/5)	15/15 (100%)	15/15 (100%)	5/14 (36%)
**BPaJ**					ND	ND	4.20 ± 0.82 (5/5)	ND	15/15 (100%)	4/15 (27%)
**BPaC**					4.32 ± 0.24 (5/5)	1.27 ± 0.72 (4/5)	1.87 ± 1.14 (4/5)	10/15 (67%)	4/15 (27%)	0/15 (0%)
**BPaCJ**					3.53 ± 0.24 (5/5)	0.15 ± 0.27 (1/4)	0.77 ± 1.09 (2/5)	5/15 (33%)	5/15 (33%)	0/15 (0%)

Supplementary Table 5: Study A, *p*-values for using the Fisher's exact test two-sided for the proportions of mice relapsing after 8 weeks of treatment with Bonferroni correction. Bedaquiline (B), pretomanid (Pa), linezolid (L), moxifloxacin (M), pyrazinamide (Z), clofazimine (C) and JNJ-2901 (J).

	BPaMZ	BPaL	BPaM	BPaMJ	BPaC	BPaCJ
BPaMZ		0.0015	0.0015	0.0015	0.003	0.6315
BPaL			>0.999	>0.999	>0.999	0.117
BPaM				>0.999	>0.999	0.3795
BPaMJ					0.6315	0.003
BPaC						>0.999
BPaCJ						

Supplementary Table 6: Study B, *p*-values for using the Fisher's exact test two-sided for the proportions of mice relapsing after 8 weeks of treatment with Bonferroni correction. Bedaquiline (B), pretomanid (Pa), linezolid (L), moxifloxacin (M), clofazimine (C) and JNJ-2901 (J).

	BPaL	BPaJ	BPaM	BPaMJ	BPaC	BPaCJ
BPaL		>0.999	>0.999	>0.999	0.0015	0.003
BPaJ			>0.999	>0.999	0.0015	0.003
BPaM				>0.999	0.0015	0.003
BPaMJ					0.0015	0.003
BPaC						>0.999
BPaCJ						

Supplementary Table 7: Study B, *p*-values using the Fisher's exact test two-sided for the proportions of mice relapsing after 12 weeks of treatment with Bonferroni correction. Bedaquiline (B), pretomanid (Pa), linezolid (L), moxifloxacin (M), clofazimine (C) and JNJ-2901 (J).

	BPaL	BPaJ	BPaM	BPaMJ	BPaC	BPaCJ
BPaL		>0.999	>0.999	>0.999	>0.999	>0.999
BPaJ			>0.999	>0.999	>0.999	>0.999
BPaM				0.2535	>0.999	>0.999
BPaMJ					0.2535	0.2535
BPaC						>0.999
BPaCJ						

Supplementary Table 8: Lung bacterial burden and percentage of relapse in *M. tuberculosis*-infected mice (study C) - Swiss female mice were infected intravenously with 6.1 log_{10} *M. tuberculosis* H37Rv[2]. Beginning 2 weeks post-infection (pi) treatment was administered 5 days per week for 16 weeks. Lung bacterial burden was assessed 3 months after 16 weeks of treatment. Twelve weeks after 16 weeks of treatment, the proportion of mice with relapse was calculated (n=20 mice/group initially, mice missing at assessment of relapse correspond to early euthanize due to oral gavage accidents). D-13 = 1 day pi; D0 = day of treatment initiation, 2 weeks pi. Mice received different treatments combinations containing bedaquiline (B; 25 mg kg^{-1}), pretomanid (Pa; 40 mg kg^{-1}), linezolid (L; 100 mg kg^{-1}), moxifloxacin (M; 100 mg kg^{-1}) and JNJ-2901 (J; 10 mg kg^{-1}), rifampicin (R, 10 mg kg^{-1}), isoniazid (H, 25 mg kg^{-1}) and pyrazinamide (Z, 150 mg kg^{-1}). *The proportion of mice with detectable CFU among all mice analysed was determined and expressed as % relapse. CFU, colony-forming unit; D, day; SD, standard deviation; Tx, treatment; wks, weeks.

Treatment	Mean log_{10} CFU count ± SD			Relapse, n/N (%)
	D-13	D0	16 wks Tx + 12 wks	16 wks Tx + 12 wks
**Untreated**	5.73 ± 0.2	7.18 ± 03		
**RHZ**			1.36 ± 1.1	12/18 (67%)
**BPaMZ**			0.49 ± 0.9	5/19 (26%)
**BPaL**			2.06 ± 1.3	16/18 (89%)
**BPaLJ**			0.95 ± 1.1	10/20 (50%)
**BPaLM**			0.87 ± 0.7	13/17 (76%)

Supplementary Table 9: Study C, *p*-values for using the Fisher's exact test two-sided for the proportions of mice relapsing after 16 weeks of treatment with Bonferroni correction. Bedaquiline (B), pretomanid (Pa), linezolid (L), moxifloxacin (M) and JNJ-2901 (J), rifampicin (R), isoniazid (H) and pyrazinamide (Z).

	**RHZ**	**BPaMZ**	**BPaL**	**BpaLJ**	**BPaLM**
**RHZ**		0.217	>0.999	>0.999	>0.999
**BPaMZ**			0.002	>0.999	>0.067
**BPaL**				>0.149	>0.999
**BPaLJ**					>0.999
**BPaLM**					

Supplementary Table 10: Study C, *p*-values using the Kruskal-Wallis test for the lung CFUs of mice relapsing after 16 weeks of treatment with adjustment for multiple comparisons (Dunn's test). Bedaquiline

(B), pretomanid (Pa), linezolid (L), moxifloxacin (M) and JNJ-2901 (J), rifampicin (R), isoniazid (H) and pyrazinamide (Z).

	RHZ	BPaMZ	BPaL	BpaLJ	BPaLM
**RHZ**		>0.05	>0.05	>0.05	>0.05
**BPaMZ**			0.0003	>0.05	>0.05
**BPaL**				0.04	>0.05
**BPaLJ**					>0.05
**BPaLM**					

Supplementary Table 11: Lung bacterial burden during treatment and relapse period in *M. tuberculosis*-infected mice (Study D) - Four to 6 weeks old BALB/c female mice were infected by high-dose aerosol with *M. tuberculosis* H37Rv. Treatment was administered from 2 weeks post-infection (pi), 5 days per week for 8 to 16 weeks (8-week initial phase of BPaL+/- 8-week continuation phase). Mice were held for up to 32 weeks after treatment initiation to determine the proportion of relapse after treatment cessation. Mice received different treatment combinations containing bedaquiline (B; 25 mg kg^{-1}), pretomanid (Pa; 100 mg kg^{-1}), linezolid (L; 100 mg kg^{-1}) and JNJ-2901 (J; 5 mg kg^{-1}). *Bactericidal activity after the first 8 week period of treatment. **The BPaL/none group did not receive any treatment after the initial 8 weeks of BPaL. N, total number of mice/group; n, number of mice with CFU at plating. CFU, colony-forming unit; D, day; SD, standard deviation; wks, weeks.

Treatment	Mean ± SD log_{10} CFU/lung (relapse, n/N)						Relapse, n/N (%)
	-2 wks	Day 0	2 wks	8 wks	12 wks	16 wks	16 wks (+ 16 wks)
**Untreated**	3.68±0.22	7.20±0.13	9.10±0.50				
**BPaL***				0.11±0.24			
**BPaL/BPa**					0.07±0.16 (1/5)	0.00±0.00 (0/5)	0/15 (0%)
**BPaL/none****					0.32±0.20 (4/5)	0.63±1.18 (2/5)	4/15 (27%)
**BPaL/BJ**					0.00±0.00 (0/5)	0.00±0.00 (0/4)	0/15 (0%)
**BPaL/B**					0.07±0.16 (1/5)	0.25±0.24 (3/5)	0/15 (0%)

Supplementary Table 12: Lung bacterial burden and percentage of relapse in *M. tuberculosis*-infected mice (Study E) - Six to 8 weeks old BALB/c female mice were infected by high-dose aerosol with *M. tuberculosis* Erdman. Treatment was administered from 11 days post-infection (pi) 5 days per week for 4, 6, 8, 12, 16 and 20 weeks. 12 weeks after treatment cessation (+12 wks), the proportion of relapse was calculated (n=15 mice). n= number of mice with CFU at plating/total. D-10 = 1 day pi; D0 = day of treatment initiation, 11 days pi. *One mouse from the indicated groups reached humane end points and was euthanised before the planned date. Gavage errors are suspected. **One mouse reached humane end points and was euthanised before the planned date owing to cage-related trauma. ***Two mice reached humane end points and were euthanised before the planned date, one with an injury-related leg mass and one due to weight loss of unknown cause. Mice received different treatment combinations containing bedaquiline (B; 25 mg kg^{-1}), clofazimine (C; 20 mg kg^{-1}), pyrazinamide (Z; 150 mg kg^{-1}), isoniazid (10 mg kg^{-1}), rifampicin (10 mg kg^{-1}), and ethambutol (100 mg kg^{-1}) and Telacebec (T; 10 mg kg^{-1}). N, total number of mice/groups; n, number of mice with CFU at plating. CFU, colony-forming unit; D, days; M, months; SEM, standard error of the mean, SOC, standard of care.

	**Mean ± SEM log_{10} CFU $lung^{-1}$ (Relapse, n/N)**			**Relapse, n/N (%)**					
**Treatment**	D-10	D0	8 wks	4 wks (+12 wks)	6 wks (+12 wks)	8 wks (+12 wks)	12 wks (+12 wks)	16 wks (+12 wks)	20 wks (+12 wks)
**Untreated**	4.17 ± 0.02	7.02 ± 0.02							
**CZ**			2.63 ± 0.15 (5/5)				14/14* (100%)	15/15 (100%)	
**CZT**			<0.40 ± 0.00 (1/5)			4/15 (27%)	0/15 (0%)	0/14** (0%)	
**BCZ**			<0.40 ± 0.00 (0/5)	9/15 (60%)	0/15 (0%)	0/14* (0%)			
**BCZT**			<0.40 ± 0.00 (0/5)	5/14* (36%)	0/14* (0%)	0/15 (0%)			
**HRZE (SOC)**			2.39 ± 0.13 (5/5)					10/15 (67%)	1/13*** (8%)

Supplementary Table 13: Study E, *P*-values using the Fisher's exact test two-sided for the proportions of mice relapsing 12 weeks after the indicated treatment time - B, bedaquiline; T, telacebec; C, clofazimine; E, ethambutol; H, isoniazid; L, linezolid; NA, not applicable; Pa, pretomanid; R, rifampicin; Z, pyrazinamide.

Regimens/ Treatment duration in months		CZ		CZT			BCZ			BCZT			HRZE	
		12 wks	16 wks	8 wks	12 wks	16 wks	4 wks	6 wks	8 wks	4 wks	6 wks	8 wks	16 wks	20 wks
CZ	12 wks	NA	>0.9999	<0.0001	<0.0001	<0.0001	0.0407	<0.0001	<0.0001	0.0006	<0.0001	<0.0001	0.0421	<0.0001
	16 wks		NA	<0.0001	<0.0001	<0.0001	0.0169	<0.0001	<0.0001	0.0002	<0.0001	<0.0001	0.0421	<0.0001
CZT	8 wks			NA	0.0996	0.0996	0.0656	0.0996	0.0996	0.6999	0.0996	0.0996	0.0656	0.3333
	12 wks				NA	>0.9999	0.0002	>0.9999	>0.9999	0.0169	>0.9999	>0.9999	0.0002	0.4643
	16 wks					NA	0.0006	>0.9999	>0.9999	0.0407	>0.9999	>0.9999	0.0002	0.4815
BCZ	4 wks						NA	0.0002	0.0006	0.2568	0.0006	0.0002	>0.9999	0.0044
	6 wks							NA	>0.9999	0.0169	>0.9999	>0.9999	0.0002	0.4643
	8 wks								NA	0.0407	>0.9999	>0.9999	0.0002	0.4815
BCZT	4 wks									NA	0.0407	0.0169	0.1431	0.1647
	6 wks										NA	>0.9999	0.0002	0.4815
	8 wks											NA	0.0002	0.4643
HRZE	16 wks												NA	0.0021
	20 wks													NA

Supplementary Table 14: Clinical isolate susceptibility to JNJ-4052 and bedaquiline (BDQ)

Strain Number	Source	Lineage	Isoniazid Susceptibility	Rifampicin Susceptibility	BDQ MIC_{90} (nM)	JNJ-4052 MIC_{90} (nM)
H37Rv	Institut Pasteur	4	S	S	197	6
N1283	Gagneux	4	S	S	104	0.73
Mtb-9271	Karonga	4	S	S	50	0.2
Mtb-7489	Karonga	1	S	S	430	2.5
Mtb-4779	Karonga	2	S	S	45	0.29
Mtb-61164	Karonga	2	S	S	188	0.79
Mtb-7496	Karonga	3	**R**	S	106	0.29
Mtb-2703	Karonga	3	S	**R**	64	0.13
Mtb-0617	Karonga	3	S	S	431	1.3
Mtb-4980	Karonga	4	S	S	63	0.7
Mtb-6787	Karonga	4	S	S	53	0.71
N0072	Gagneux	1	S	S	170	1.2
N0054	Gagneux	3	S	S	354	0.85

Supplementary methods

HepG2 Cytotoxicity Assay

To assess the cytotoxicity potential of compounds, cellular ATP content was determined, as an indicator of metabolically active cells. Briefly, HepG2 cells, grown in EMEM media, were seeded at 1000 cells/well in 384-well plates (Corning Inc., New York) and allowed to adhere for 24 hours. The HepG2 cell line (human hepatocellular carcinoma cells) was originally obtained from ATCC (HB-8065). The cells were grown, expanded and frozen (mother bank) according to the provider's instructions. Compounds were prepared as 100× concentrated stock solutions in dimethyl sulfoxide (DMSO), and the final DMSO concentration was 1%. Cells were exposed to the compounds (concentration range: 0.2–100 µM, dilution factor 2) for 72 hours before cellular ATP content was measured. Cellular ATP concentrations were assessed by using the CellTiter-Glo 2.0 Assay (Promega, Madison, WI), and the readout was performed by detecting luminescence on an EnVision 2105 Multilabel Reader (PerkinElmer, Waltham, MA). Results were imported in Genedata Screener software (Genedata, Basel, Switzerland) to create dose–response curves and calculate the IC_{20} values of each compound. Test items are strongly cytotoxic with IC_{20} value <10 µM and moderate cytotoxic with IC_{20} values between 10 and 30 µM.

Mitochondrial toxicity assay Glu/Gal

To assess the mitochondrial toxicity potential of compounds, the cytotoxicity potential (determined by ATP measurement) of the compounds was compared in glucose versus galactose culture conditions. Briefly, HepG2 cells, either grown in glucose or galactose-containing DMEM media, were seeded at 3000 cells/well in 384-well plates (Corning Inc., New York) and allowed to adhere for 24 hours. The HepG2 cell line (human hepatocellular carcinoma cells) was originally obtained from ATCC (HB-8065). The cells were grown, expanded and frozen (mother bank) according to the provider's instructions. Compounds were prepared as 100× concentrated stock solutions in dimethyl sulfoxide (DMSO), and the final DMSO concentration was 1%. Cells were exposed to the compounds (concentration range: 0.2–100 µM, dilution factor 2) for 24 hours before cellular ATP content was measured. Cellular ATP concentrations were assessed by using the CellTiter-Glo 2.0 Assay (Promega, Madison, WI), and the readout was performed by detecting luminescence on an EnVision 2105 Multilabel Reader (PerkinElmer, Waltham, MA). Results were imported in Genedata Screener software (Genedata, Basel, Switzerland) to create dose–response curves and calculate the IC50 values of each compound. Subsequently, the ratio between Glu IC50 and Gal IC50 for each compound was calculated. Compounds with a significantly higher cytotoxicity potential

in galactose-grown cells (Glu/Gal ratio ≥ 5) are determined as substances that induce mitochondrial dysfunction as the primary MoA.

Ames II Mutagenicity Assay

The AmesII Mutagenicity Assay (Xenometrix AG, Allschwil, Switzerland) is a colorimetric, high-throughput version of the original bacterial reverse mutation test. After incubation of the *Salmonella typhimurium* strains TA98 and TAMix (consisting of the strains TA7001-7006 in equal proportions) with a mutagenic product, the bromocresol pH indicator turns from purple to yellow as a consequence of bacterial growth. A modified version of this assay was used to assess the mutagenic potential of test compounds.

Fresh 3 h cultures were made using overnight cultures, a 1/4 dilution of the overnight culture with fresh growth medium. The compound concentrations (2-fold serial dilution for 500 µg/mL final concentration) and S9-mix were prepared just before use.

Approximately 10^7 histidine-negative bacteria were exposed to 8 concentrations of the test compound, as well as a positive and solvent control, in the presence and absence of S9, for 90 min at 37 °C in a medium containing sufficient histidine to support approximately two cell divisions (48-deepwell block). After incubation, 3.5 mL of pH Indicator Medium (lacking histidine) was added to each well, and subsequently, 40 µL volumes were transferred to a 384-well plate in 12-fold according to a fixed pattern. The 384-well plates were incubated in the dark at 37 °C for 48 h.

After incubation, bacteria that had undergone reversion to histidine-positive had grown into colonies. Metabolism by the bacterial colony reduces the pH of the medium, changing the color from purple to yellow in that well. A total of 96 wells were counted per concentration and condition. The number of yellow wells containing revertant colonies were counted for each concentration and compared to the concurrent solvent control. An increase in the number of revertant colonies upon exposure to the test compound relative to the concurrent controls indicates that the test compound is mutagenic in the assay.

General scheme for the synthesis of compound JNJ-2901

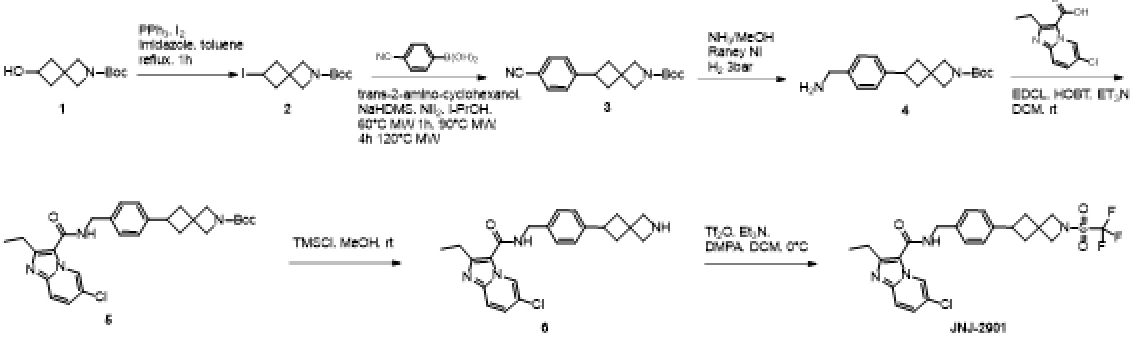

*Synthesis of intermediate tert-butyl 6-iodo-2-azaspiro[3.3]heptane-2-carboxylate (**2**)*

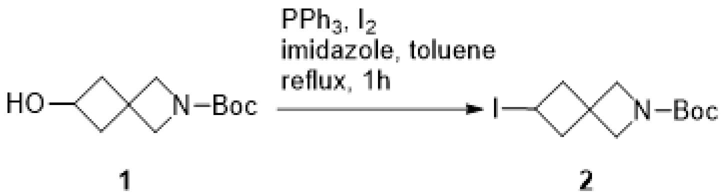

Triphenylphosphine (1.89 g, 7.20 mmol), imidazole (735 mg, 10.8 mmol) and iodine (1.37 g, 5.40 mmol) were added to a solution of tert-butyl 6-hydroxy-2-azaspiro[3.3]heptane-2-carboxylate **1** (768 mg, 3.60 mmol) in toluene (50 mL). The resulting mixture was refluxed for 1 hour. The mixture was cooled to 25°C, washed with water (100 mL) and brine (50 mL). The separated organic layer was dried, filtered and the filtrate was concentrated under vacuum. The residue was purified by flash column chromatography over silica gel (eluent: petroleum ether/ethyl acetate 1/0 to 1/1) to give tert-butyl 6-iodo-2-azaspiro[3.3]heptane-2-carboxylate **2** (1.20 g, yield: 93%).

*Synthesis of intermediate tert-butyl 6-(4-cyanophenyl)-2-azaspiro[3.3]heptane-2-carboxylate (**3**)*

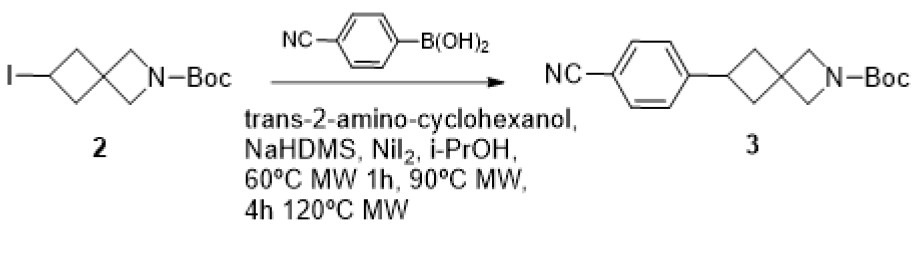

A mixture of intermediate **2** (364 mg, 2.47 mmol), trans-2-amino-cyclohexanol (28.5 mg, 0.248 mmol) and Nickel iodine (38.7 mg, 0.124 mmol) in *i*-PrOH (4 mL) was stirred at 25°C for 30 minutes under nitrogen flow. NaHMDS (2.48 mL, 1 M in THF) was added, and the mixture was stirred for 10 minutes under nitrogen flow. A solution of 4-cyanophenylboronic acid (400 mg, 1.24 mmol) in *i*-PrOH (4 mL) was added and the mixture was stirred at 60 °C under microwave for 1 hour, at 90°C for 1 hour and at 120°C for 4 hours. The mixture was diluted with

dichloromethane (50 mL), washed with water (2x50 mL) and brine (20 mL). The organic layer was dried over sodium sulfate, filtered and concentrated under vacuum. The residue was purified by column chromatography over silica gel (eluent: petroleum ether/ethyl acetate 5/1) to give tert-butyl 6-(4-cyanophenyl)-2-azaspiro[3.3]heptane-2-carboxylate **3** (300 mg, yield: 37%).

*Synthesis of intermediate tert-butyl 6-(4-(aminomethyl)phenyl)-2-azaspiro[3.3]heptane-2-carboxylate (**4**)*

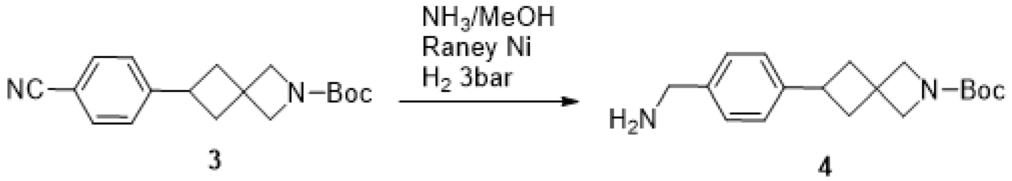

Over a solution of intermediate **3** (1.3g) in ammonia, Raney Nickel (5 equivalents) was added and the mixture stirred at room temperature under 3 bar of H_2 for 2 hours. The resulting mixture was filtered off and evaporated in vacuo, the filtrate may be taken up in EtOAc, filtered on a pad of Celite® and concentrated to provide on tert-butyl 6-(4-(aminomethyl)phenyl)-2-azaspiro[3.3]heptane-2-carboxylate **4** (1.29g, yield 81%).

*Synthesis of intermediate tert-butyl 6-(4-((6-chloro-2-ethylimidazo[1,2-a]pyridine-3-carboxamido) methyl)phenyl)-2-azaspiro[3.3]heptane-2-carboxylate (**5**)*

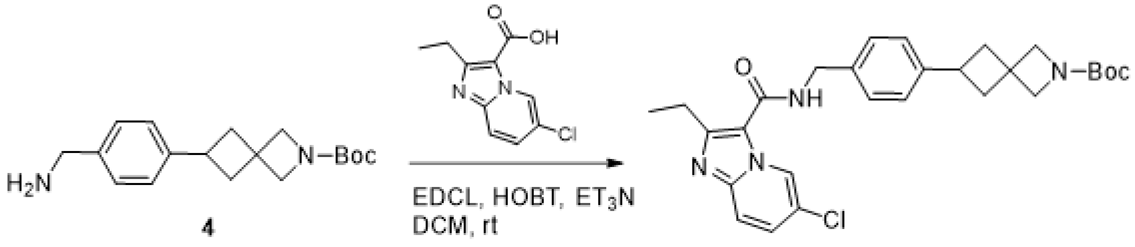

To a solution of 6-chloro-2-ethylimidazo[1,2-a]pyridine-3-carboxylic acid (0.117 g, 0.504 mmol) in DCM (5.1 mL) and triethylamine (0.18 mL) were added EDCI (145 mg, 0.756 mmol) and HOBt (103 mg, 0.760 mmol) and the mixture was stirred at room temperature for 30 min. Intermediate **4** (0.162 g, 0.536 mmol) was added and the mixture was stirred at room temperature for 4 h. The mixture was washed with water (2x). The organic layer was dried over $MgSO_4$, filtered and evaporated to dryness to give 0.293 g of tert-butyl 6-(4-((6-chloro-2-ethylimidazo[1,2-a]pyridine-3-carboxamido) methyl)phenyl)-2-azaspiro[3.3] heptane-2-carboxylate **5** as colourless oil (quant.), used as such in the next step.

*Synthesis of intermediate N-(4-(2-azaspiro[3.3]heptan-6-yl)benzyl)-6-chloro-2-ethylimidazo[1,2-a]pyridine-3-carboxamide (**6**)*

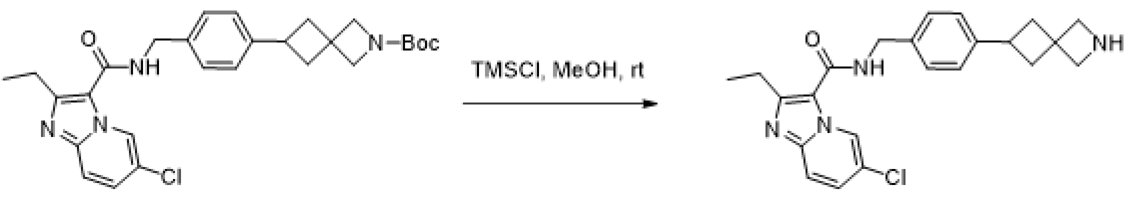

5 6

To a solution of intermediate **5** (0.291 g, 0.572 mmol) in methanol (5.9 mL) was added trimethylchlorosilane (0.37 mL, 2.94 mmol) and the mixture was stirred at room temperature for 16 h. The mixture was evaporated to dryness to give N-(4-(2-azaspiro[3.3]heptan-6-yl)benzyl)-6-chloro-2-ethylimidazo[1,2-a]pyridine-3-carboxamide (6) 0.304 g as a pale yellow foam (quant.).

*Synthesis of 6-chloro-2-ethyl-N-(4-(2-((trifluoromethyl)sulfonyl)-2-azaspiro[3.3]heptan-6-yl)benzyl)imidazo[1,2-a]pyridine-3-carboxamide (**JNJ-2901**)*

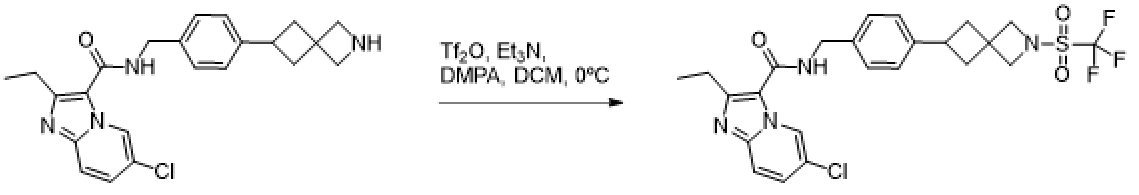

6 **JNJ-2901**

Trifluoromethanesulfonic anhydride (0.12 mL, 0.696 mmol) was added to a solution of intermediate **6** (155 mg, 0.348 mmol) and DMAP (2.13 mg, 17.4 µmol) in triethylamine (0.39 mL, 2.78 mmol) and DCM (5.3 mL) at 0 °C. The resulting mixture was stirred at 0 °C for 6 h. Water was added and the organic layer was washed with water, dried over $MgSO_4$, filtered and evaporated to dryness. The crude product was purified by preparative LC (irregular SiOH, 15-40 µm, 40 g, Grace, dry loading (silica), mobile phase gradient Heptane/EtOAc from 90/10 to 10/90) to obtain 186 mg of a pale yellow solid, which was triturated in heptane and purified by preparative LC (spherical C18 25 µm, 40 g YMC-ODS-25, dry loading (Celite®), mobile phase gradient: 0.2% aq. NH_4HCO_3/MeCN from 90/10 to 0/100) to give 0.112 g of 6-chloro-2-ethyl-N-(4-(2-((trifluoromethyl)sulfonyl)-2-azaspiro[3.3]heptan-6-yl)benzyl)imidazo[1,2-a]pyridine-3-carboxamide (JNJ-2901) as a white solid (59%). 1H NMR (500 MHz, DMSO-d^6) δ ppm 9.07 (dd, *J*=2.1, 0.7 Hz, 1 H) 8.46 (t, *J*=6.1 Hz, 1 H) 7.66 (dd, *J*=9.5, 0.9 Hz, 1 H) 7.45 (dd, *J*=9.5, 2.1 Hz, 1 H) 7.30 (m, *J*=8.1 Hz, 2 H) 7.19 (m, *J*=8.1 Hz, 2 H) 4.49 (d, *J*=5.9 Hz, 2 H) 4.41 (s, 2 H) 4.18 (s, 2 H) 3.34 - 3.39 (m, 1 H) 2.98 (q, *J*=7.5 Hz, 2 H) 2.57 - 2.65 (m, 2 H) 2.28 - 2.35 (m, 2 H) 1.26 (t, *J*=7.5 Hz, 3 H). 13C NMR (125.76 MHz, DMSO-d6) δ ppm 160.99 (s, 1 C) 151.51 (s, 1 C) 143.77 (s, 1 C) 143.30 (s, 1 C) 137.60 (s, 1 C) 127.80 (s, 2 C) 127.52

(s, 1 C) 126.77 (s, 2 C) 125.29 (s, 1 C) 120.13 (s, 1 C) 117.65 (s, 1 C) 120.62 (q, *J*=323.00 Hz, 1 C) 116.20 (s, 1 C) 65.25 (s, 1 C) 63.28 (d, *J*=4.60 Hz, 1 C) 42.64 (s, 1 C) 39.70 (s, 2 C) 34.29 (s, 1 C) 33.34 (s, 1 C) 22.32 (s, 1 C) 13.54 (s, 1 C).

General scheme for the synthesis of compound JNJ-4052

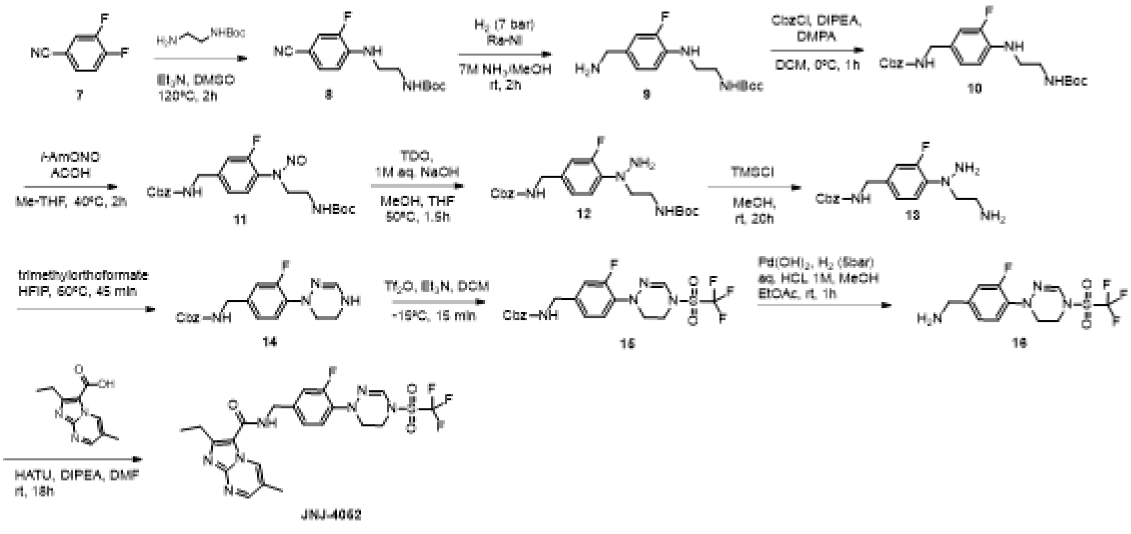

*<u>Synthesis of intermediate tert-butyl (2-((4-cyano-2-fluorophenyl)amino)ethyl)carbamate (**8**)</u>*

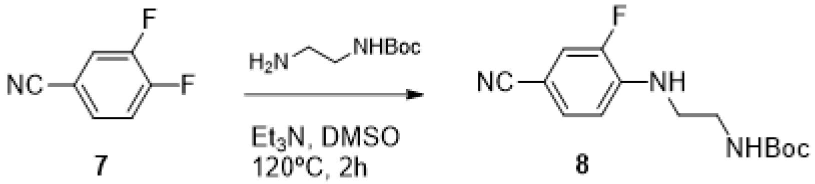

A mixture of 3,4-difluorobenzonitrile (3.67 g, 26.4 mmol), N-Boc-1,2-diaminoethane (5.50 g, 34.3 mmol) and Et_3N (14.7 mL, 105 mmol) in DMSO (47 mL) was stirred at 120 °C for 2 h. The reaction mixture was cooled down and diluted with EtOAc and water. The layers were separated, and the aqueous phase was extracted with EtOAc (twice). The combined organic layers were washed with brine (3 times), dried over $MgSO_4$, filtered, and evaporated in vacuo. The residue was purified by preparative LC (irregular SiOH 15-40 µm, 80 g, liquid injection (DCM), mobile phase: heptane/EtOAc, gradient from 100:0 to 50:50) to give 5.02 g of intermediate tert-butyl (2-((4-cyano-2-fluorophenyl)amino)ethyl) carbamate (**8**) as a white solid (68%).

*<u>Synthesis of intermediate tert-butyl (2-((4-(aminomethyl)-2-fluorophenyl)amino)ethyl) carbamate (**9**)</u>*

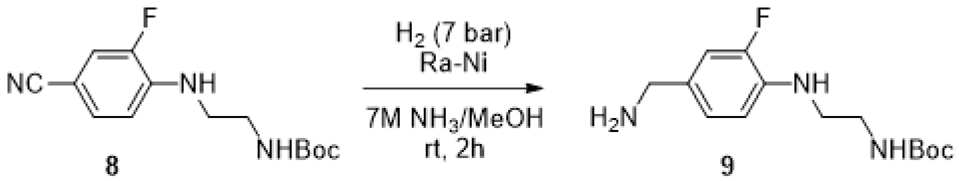

In an autoclave, to a solution of intermediate **8** (2.00 g, 7.16 mmol) in a 7M solution of NH_3 in MeOH (70 mL), purged with nitrogen, was added Raney-Nickel (3.39 g, 57.7 mmol). The reaction mixture was hydrogenated under 7 bars at room temperature for 2 h. The mixture was filtered through a pad of Celite® and rinsed with MeOH. The filtrate was concentrated in vacuo to give 2.11 g of intermediate tert-butyl (2-((4-(aminomethyl)-2-fluorophenyl)amino)ethyl) carbamate (**9**) as a white solid (Quant.).

*Synthesis of intermediate tert-butyl (2-((4-((((benzyloxy)carbonyl)amino)methyl)-2-fluorophenyl)amino)ethyl)carbamate (**10**)*

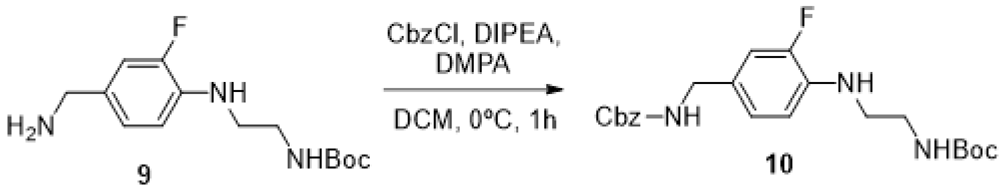

Under N_2 at 0°C, benzylchloroformate (1.06 mL, 7.41 mmol) was added dropwise over 10 min to a mixture of intermediate **9** (2 g, 7.06 mmol) and DIPEA (1.40 mL, 8.12 mmol) in DCM (26 mL). The reaction mixture was stirred at 0 °C for 1h. Then water was added, and the reaction mixture was stirred at room temperature for 10 min. The organic layer was separated, and the aqueous layer was extracted with DCM (once). The combined organic extracts were washed with $NaHCO_3$ (sat., aq.), dried over $MgSO_4$, filtered and the solvent was removed under reduced pressure to give 2.53 g of intermediate tert-butyl (2-((4-((((benzyloxy)carbonyl)amino)methyl)-2-fluorophenyl)amino)ethyl)carbamate (**10**) as an off-white solid (86%).

*Synthesis of intermediate tert-butyl (2-((4-((((benzyloxy)carbonyl)amino)methyl)-2-fluorophenyl)(nitroso)amino)ethyl)carbamate (**11**)*

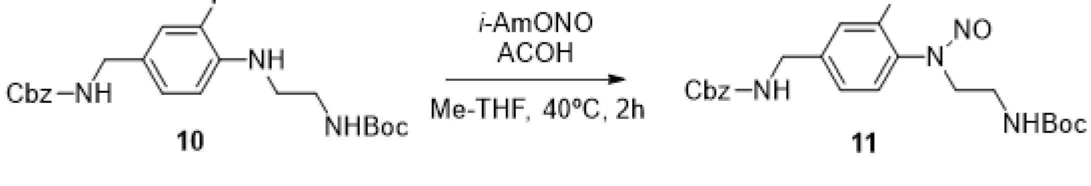

Intermediate **10** (2.53 g, 6.06 mmol) was solubilized at 40 °C in Me-THF (46 mL) and AcOH (3.5 mL). Isopentylnitrite (4.07 mL, 30.3 mmol) was added dropwise, and the reaction mixture was stirred at 40 °C for 2 h. The solution was diluted with EtOAc and $NaHCO_3$ (sat., aq.). The

layers were separated, and the organic phase was, dried over $MgSO_4$ and evaporated in vacuo to give 3.2 g of intermediate tert-butyl (2-((4-((((benzyloxy) carbonyl)amino)methyl)-2-fluorophenyl)(nitroso)amino)ethyl)carbamate (**11**) as a yellow oil used in next step without any further purification.

*Synthesis of intermediate tert-butyl (2-(1-(4-((((benzyloxy)carbonyl)amino)methyl)-2-fluorophenyl)hydrazineyl)ethyl)carbamate (**12**)*

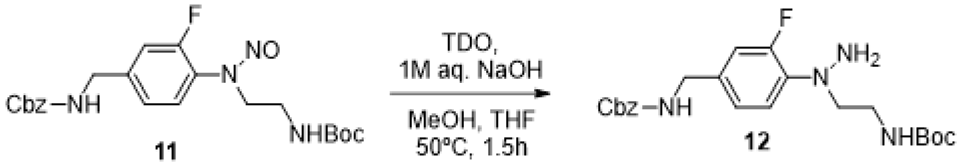

A solution of intermediate **11** (6.06 mmol theoretical) in THF (61 mL) and MeOH (39 mL) was treated with NaOH (1M, aq., 29 mL). Thiourea dioxide (formamidinesulfonic acid) (3.28 g, 30.3 mmol) was then added, and the reaction mixture was stirred at 50 °C for 1.5 h. The reaction mixture was diluted with EtOAc and K_2CO_3 (10%, aq.) was added. The layers were separated. The aqueous layer was extracted with EtOAc (once)). The combined organic layers were dried over $MgSO_4$, filtered, and evaporated in vacuo. The crude (m= 3.46 g) was purified by preparative LC (irregular SiOH, 40 mm, 40 g, mobile phase gradient: Heptane/EtOAc 90/10 to 20/80). The fractions containing product were combined and evaporated under vacuum to give 2.22 g of intermediate of intermediate tert-butyl (2-(1-(4-((((benzyloxy)carbonyl)amino)methyl)-2-fluorophenyl)hydrazineyl)ethyl) carbamate (**12**) as a yellow oil (84 % over two steps).

*Synthesis of intermediate benzyl (4-(1-(2-aminoethyl)hydrazineyl)-3-fluorobenzyl) carbamate (**13**)*

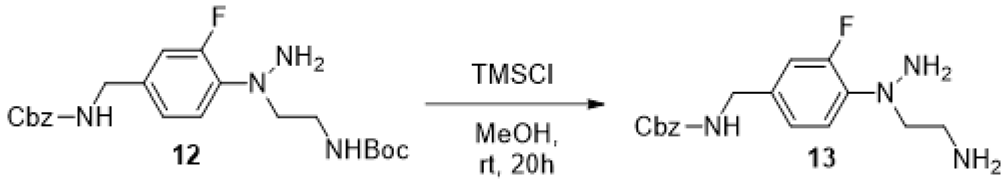

To a solution of intermediate **12** (2.22 g, 5.13 mmol) in MeOH (52 mL) was added dropwise TMSCl (5.2 mL, 41 mmol). The reaction mixture was stirred at room temperature for 20 hours and concentrated in vacuo. Et_2O was added to the residue and the gum was triturated. The solvent was removed under reduced pressure to give 2.06 g of intermediate benzyl (4-(1-(2-aminoethyl)hydrazineyl)-3-fluorobenzyl) carbamate (**13**) as a pale green solid (99%).

*Synthesis of intermediate benzyl (4-(5,6-dihydro-1,2,4-triazin-1(4H)-yl)-3-fluorobenzyl) carbamate (**14**)*

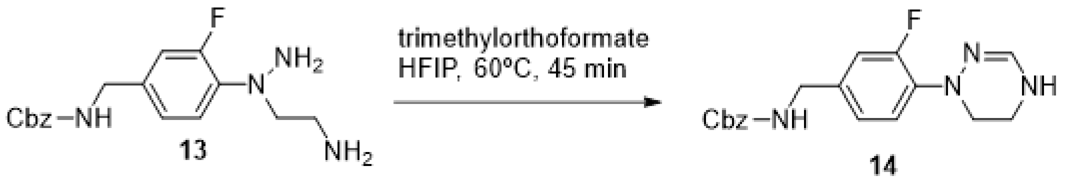

A solution of intermediate **13** (19.6 g, 48.4 mmol) and Trimethylorthoformate (15.9 mL, 145 mmol) in HFIP (490 mL) was stirred at 60 °C for 45 min. The reaction mixture was evaporated. The residue was diluted in DCM and a 10 % aq. solution of K_2CO_3 was added. The aqueous layer was extracted twice with DCM/MeOH (95/5). 20 The combined organic layers were dried on $MgSO_4$, filtered off and evaporated. The crude (m=25.6 g) was purified by preparative LC (regular SiOH 30 pm, 330 g, dry loading (celite®), mobile phase gradient: from Heptane 75%, EtOAc/MeOH (9:1) 25% to Heptane 25%, EtOAc/MeOH (9:1). Fractions containing product were combined and evaporated to give 14.61 g of intermediate benzyl (4-(5,6-dihydro-1,2,4-triazin-1(4H)-yl)-3-fluorobenzyl) carbamate (**14**) as a colorless oil which crystallized on standing (85%).

*Synthesis of intermediate benzyl (3-fluoro-4-(4-((trifluoromethyl)sulfonyl)-5,6-dihydro-1,2,4-triazin-1(4H)-yl)benzyl)carbamate (**15**)*

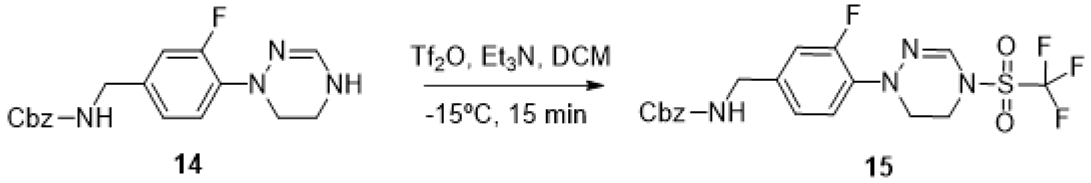

To a solution of intermediate **14** (14.6 g, 42.7 mmol) and DIPE (22.1 mL, 128 mmol) in dry DCM (340 mL) at -5 °C (ice/NaCl solid) was added dropwise Tf_2O 1M in DCM (47 mL, 47 mmol) over 15 min using a dropping funnel and stirring was continued for 5 min. The reaction mixture was quenched with a saturated aqueous solution of $NaHCO_3$. The layers were separated, and the aqueous layer was extracted with DCM (twice). The combined organic layer was dried over $MgSO_4$, filtered off and concentrated. The crude (m= 36.4 g) was purified by preparative LC (regular SiOH, 30 pm, 120 g, dry loading (celite®), mobile phase gradient: Heptane/EtOAc 90/10 to 70/30). The fractions containing product were combined and evaporated under vacuum to give 10.18 g of intermediate benzyl (3-fluoro-4-(4-((trifluoromethyl)sulfonyl)-5,6-dihydro-1,2,4-triazin-1(4H)-yl)benzyl)carbamate (**15**) as a white solid (50%).

*Synthesis of intermediate (3-fluoro-4-(4-((trifluoromethyl)sulfonyl)-5,6-dihydro-1,2,4-triazin-1(4H)-yl)phenyl)methanamine (**16**)*

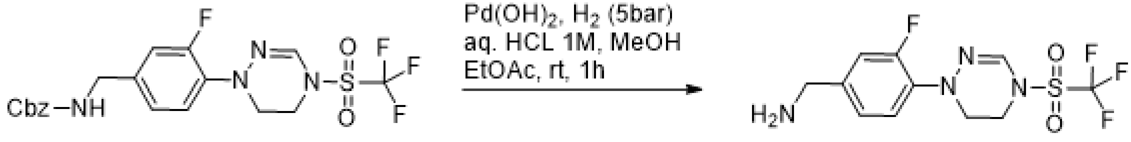

15 **16**

In a steal bomb, a mixture of intermediate **15** (10.2 g, 21.5 mmol), Palladium 20 hydroxide 20% on carbon nominally 50% water (3.01 g, 2.15 mmol) and aqueous HCl 3M (7.15 mL, 7.15 mmol) in MeOH (150 mL) and EtOAc (150 mL) was hydrogenated under 5 bars of H_2 at room temperature for 1 h. The mixture was filtered on a pad of celite® and washed with MeOH. The filtrate was evaporated then co-evaporated with MeOH (twice) to give 7.86 g of intermediate (3-fluoro-4-(4-((trifluoromethyl)sulfonyl)-5,6-dihydro-1,2,4-triazin-1(4H)-yl)phenyl)methanamine (**16**).

*Synthesis of 2-ethyl-N-(3-fluoro-4-(4-((trifluoromethyl)sulfonyl)-5,6-dihydro-1,2,4-triazin-1(4H)-yl)benzyl)-6-methylimidazo[1,2-a]pyrimidine-3-carboxamide (**JnJ-4052**)*

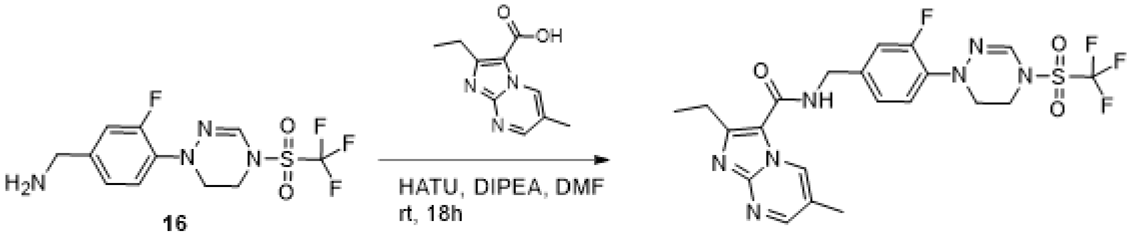

16 **JNJ-4052**

A mixture of 2-ethyl-6-methylimidazo[1,2-a]pyrimidine-3-carboxylic acid[i] (190 mg, 0.518 mmol), HATU (280 mg, 0.736 mmol), DIPEA (0.163 mL, 0.958 mmol) and DMF (2.5 mL) was stirred at room temperature for 15 min then intermediate **16** (180 mg, 0.473 mmol) was added and stirring was continued over 3 days. DMF was evaporated. The residue was taken-up in DCM and water then washed with a saturated aqueous solution of $NaHCO_3$ (twice), brine (twice), dried over $MgSO_4$, filtered off and concentrated. The crude (m= 378 mg) was purified by preparative LC (regular SiOH, 30 µm, 24 g, mobile phase gradient: from Heptane 85%, EtOAc/MeOH (9:1) 15% to Heptane 25%, EtOAc/MeOH (9:1) 75). Fractions containing product were combined and concentrated to afford 277 mg as a white solid. The solid was recrystallized from EtOAc, filtered off and dried under high vacuum to afford 162 mg of 2-ethyl-N-(3-fluoro-4-(4-((trifluoromethyl)sulfonyl)-5,6-dihydro-1,2,4-triazin-1(4H)-yl)benzyl)-6-methylimidazo[1,2-a]pyrimidine-3-carboxamide (**JnJ-4052**) as a white solid (54%). 1H NMR (500 MHz, DMSO-d6) δ ppm 9.15 (dd, *J*=2.4, 1.1 Hz, 1 H) 8.51 (d, *J*=2.5 Hz, 1 H) 8.45 (t, *J*=5.9 Hz, 1 H) 7.38 (s, 1 H) 7.34 (t, *J*=8.6 Hz, 1 H) 7.24 (dd, *J*=13.2, 1.8 Hz, 1 H) 7.19 (dd, *J*=8.4, 1.8 Hz, 1 H) 4.50 (d, *J*=5.9 Hz, 2 H) 4.10 (t, *J*=4.6 Hz, 2 H) 3.62 - 3.68 (m, 2 H) 3.01 (q, *J*=7.5 Hz, 2 H) 2.34 (d, *J*=0.8 Hz, 3 H) 1.28 (t, *J*=7.5 Hz, 3 H). 13C NMR (125.76 MHz, DMSO-

d6) δ ppm 160.99 (s, 1 C) 151.51 (s, 1 C) 143.77 (s, 1 C) 143.30 (s, 1 C) 137.60 (s, 1 C) 127.80 (s, 2 C) 127.52 (s, 1 C) 126.77 (s, 2 C) 125.29 (s, 1 C) 120.13 (s, 1 C) 117.65 (s, 1 C) 120.62 (q, J=323.00 Hz, 1 C) 116.20 (s, 1 C) 65.25 (s, 1 C) 63.28 (d, J=4.60 Hz, 1 C) 42.64 (s, 1 C) 39.70 (s, 2 C) 34.29 (s, 1 C) 33.34 (s, 1 C) 22.32 (s, 1 C) 13.54 (s, 1 C)

NMR spectra are shown in Supplementary Fig. 3.

References

1. Pethe K*, et al.* Discovery of Q203, a potent clinical candidate for the treatment of tuberculosis. *Nature Medicine* **19**, 1157-1160 (2013).

2. Kort F*, et al.* Fully weekly antituberculosis regimen: a proof-of-concept study. *Eur Respir J* **56**, (2020).
