# Supplementary material for: The role of cytochrome bc1 inhibitors in future tuberculosis treatment regimens
Source: Nat Commun. 2025 Oct 22;16:9344. doi: 10.1038/s41467-025-64427-6 (PMC12546632; doi:10.1038/s41467-025-64427-6)
Supplement: Supplementary file 2 — Description of Additional Supplementary Files [file 41467_2025_64427_MOESM2_ESM.docx]

**Description of Additional Supplementary Files**

**Supplementary Data 1: Lung bactericidal treatment statistics for study E (one way ANOVA)** - B, bedaquiline; T, telacebec; C, clofazimine; E, ethambutol; H, isoniazid; R, rifampicin; Z, pyrazinamide; wks: weeks.
