# Supplementary material for: The role of cytochrome bc1 inhibitors in future tuberculosis treatment regimens
Source: Nat Commun. 2025 Oct 22;16:9344. doi: 10.1038/s41467-025-64427-6 (PMC12546632; doi:10.1038/s41467-025-64427-6)
Supplement: Supplementary file 4 — Reporting Summary [file 41467_2025_64427_MOESM4_ESM.pdf]

nature portfolio

Corresponding author(s): Clara Aguilar Perez
Dirk A. Lamprecht

Last updated by author(s): Oct 28, 2024

Reporting Summary

Nature Portfolio wishes to improve the reproducibility of the work that we publish. This form provides structure for consistency and transparency in reporting. For further information on Nature Portfolio policies, see our Editorial Policies and the Editorial Policy Checklist.

Statistics

For all statistical analyses, confirm that the following items are present in the figure legend, table legend, main text, or Methods section.

n/a	Confirmed	
☐	☒	The exact sample size (n) for each experimental group/condition, given as a discrete number and unit of measurement
☐	☒	A statement on whether measurements were taken from distinct samples or whether the same sample was measured repeatedly
☐	☒	The statistical test(s) used AND whether they are one- or two-sided<br>*Only common tests should be described solely by name; describe more complex techniques in the Methods section.*
☒	☐	A description of all covariates tested
☐	☒	A description of any assumptions or corrections, such as tests of normality and adjustment for multiple comparisons
☐	☒	A full description of the statistical parameters including central tendency (e.g. means) or other basic estimates (e.g. regression coefficient) AND variation (e.g. standard deviation) or associated estimates of uncertainty (e.g. confidence intervals)
☒	☐	For null hypothesis testing, the test statistic (e.g. F, t, r) with confidence intervals, effect sizes, degrees of freedom and P value noted<br>*Give P values as exact values whenever suitable.*
☒	☐	For Bayesian analysis, information on the choice of priors and Markov chain Monte Carlo settings
☒	☐	For hierarchical and complex designs, identification of the appropriate level for tests and full reporting of outcomes
☒	☐	Estimates of effect sizes (e.g. Cohen's d, Pearson's r), indicating how they were calculated

Our web collection on statistics for biologists contains articles on many of the points above.

Software and code

Policy information about availability of computer code

Data collection	Microsoft Excel, Perkin Elmer EnVision , SCAN-1200 counter (Interscience), Illumina NextSeq 550, Waters TargetLynx (chromatography).
Data analysis	GraphPad Prism 10, Genedata Screener, SCAN-1200 counter (Interscience), CLC Genomics Workbench v21.0.5, Waters TargetLynx (chromatography), Phoenix (Certara).

For manuscripts utilizing custom algorithms or software that are central to the research but not yet described in published literature, software must be made available to editors and reviewers. We strongly encourage code deposition in a community repository (e.g. GitHub). See the Nature Portfolio guidelines for submitting code & software for further information.

Data

Policy information about availability of data

All manuscripts must include a data availability statement. This statement should provide the following information, where applicable:

- Accession codes, unique identifiers, or web links for publicly available datasets
- A description of any restrictions on data availability
- For clinical datasets or third party data, please ensure that the statement adheres to our policy

All data generated or analysed during this study are included in this published article (and its supplementary information files). Should any raw data files be needed in another format they are available from the corresponding author upon reasonable request. The authors confirm that all unique materials used in this manuscript are readily available from the authors.

Human research participants

Policy information about studies involving human research participants and Sex and Gender in Research.

Reporting on sex and gender	*Use the terms sex (biological attribute) and gender (shaped by social and cultural circumstances) carefully in order to avoid confusing both terms. Indicate if findings apply to only one sex or gender; describe whether sex and gender were considered in study design whether sex and/or gender was determined based on self-reporting or assigned and methods used. Provide in the source data disaggregated sex and gender data where this information has been collected, and consent has been obtained for sharing of individual-level data; provide overall numbers in this Reporting Summary. Please state if this information has not been collected. Report sex- and gender-based analyses where performed, justify reasons for lack of sex- and gender-based analysis.*
Population characteristics	*Describe the covariate-relevant population characteristics of the human research participants (e.g. age, genotypic information, past and current diagnosis and treatment categories). If you filled out the behavioural & social sciences study design questions and have nothing to add here, write "See above."*
Recruitment	*Describe how participants were recruited. Outline any potential self-selection bias or other biases that may be present and how these are likely to impact results.*
Ethics oversight	*Identify the organization(s) that approved the study protocol.*

Note that full information on the approval of the study protocol must also be provided in the manuscript.

Field-specific reporting

Please select the one below that is the best fit for your research. If you are not sure, read the appropriate sections before making your selection.

☒ Life sciences ☐ Behavioural & social sciences ☐ Ecological, evolutionary & environmental sciences

For a reference copy of the document with all sections, see nature.com/documents/nr-reporting-summary-flat.pdf

Life sciences study design

All studies must disclose on these points even when the disclosure is negative.

Sample size	For in vivo studies, various scenarios were evaluated for a sample size of 3to 15 animals per group. From the power analysis, it was concluded that considering 6 animals per group provided more than 80% power to detect all significant effects of 1.5 CFU (log10), assuming SD = 0.5, with 20 or less groups (including the reference group). For proportions large
Data exclusions	There were no data exclusions.
Replication	The in vivo efficacy studies were performed once - in cases where overlap of conditions occurred, both results were reported to show biological variability. All in vitro studies were performed at least twice, with the same result replicated in all experiments.
Randomization	Mice were infected then randomly assigned different treatment groups.
Blinding	NA

Reporting for specific materials, systems and methods

We require information from authors about some types of materials, experimental systems and methods used in many studies. Here, indicate whether each material, system or method listed is relevant to your study. If you are not sure if a list item applies to your research, read the appropriate section before selecting a response.

Materials & experimental systems

n/a	Involved in the study	
☒	☐	Antibodies
☐	☒	Eukaryotic cell lines
☒	☐	Palaeontology and archaeology
☐	☒	Animals and other organisms
☒	☐	Clinical data
☒	☐	Dual use research of concern

Methods

n/a	Involved in the study	
☒	☐	ChIP-seq
☒	☐	Flow cytometry
☒	☐	MRI-based neuroimaging

Eukaryotic cell lines

Policy information about cell lines and Sex and Gender in Research

Cell line source(s)	HepG2 (ATCC HB-8065) cells
Authentication	None of the cell lines used were authenticated.
Mycoplasma contamination	All cell lines tested negative for mycoplasma contamination.
Commonly misidentified lines (See ICLAC register)	*Name any commonly misidentified cell lines used in the study and provide a rationale for their use.*

Animals and other research organisms

Policy information about studies involving animals; ARRIVE guidelines recommended for reporting animal research, and Sex and Gender in Research

Laboratory animals	Balb/cJRj and Swiss female mice 6 to 8 weeks old
Wild animals	The study did not involve wild animals
Reporting on sex	Findings apply to mice of one sex (female), sex was not considered in the study design since it would have no impact on the results of our experiments. No additional information was collected.
Field-collected samples	The study did not involve samples collected from the field
Ethics oversight	In Studies A and B, animal experiments were performed under the European Union Directive and with local ethical committee clearance. The study procedures were reviewed by the Evotec France Ethical Committee and authorised by the French Ministry of Education Advanced Studies and Research. For Study C, the experimental project was favourably evaluated by the ethics committee n°005 Charles Darwin localised at the Pitié-Salpêtrière Hospital and clearance was given by the French Ministry of Education and Research under the number APAFIS#12380-2017112809414820 v3. The animal facility received the authorisation to carry out animal experiments (license number C-75-13-08). For Study D, ethics oversight was provided by the Johns Hopkins University Animal Care and Use Committee, which is PHS assured, USDA registered, and AAALAC accredited. For Study E, ethics oversight was provided by the Colorado State University Animal Care and Use Programme (reference number, KP 5172) which is PHS assured, USDA registered, and AAALAC accredited. For Study F, animal procedures were performed under UK Home Office project license P6CA9EB8D and approved by the London School of Hygiene & Tropical Medicine Animal Welfare Ethical Review Board. All work was conducted in accordance with the UK Animal Scientific Procedure Act (ASPA) 1986.

Note that full information on the approval of the study protocol must also be provided in the manuscript.
